# Glutamate concentrations related to depression and mania symptoms in patients with Graves' disease: A 1H‐magnetic resonance spectroscopy study

**DOI:** 10.1002/kjm2.12854

**Published:** 2024-05-31

**Authors:** Shih‐Hsien Lin, Po See Chen, Shih‐Ming Huang, Yen Kuang Yang

**Affiliations:** ^1^ Department of Psychiatry National Cheng Kung University Hospital, College of Medicine, National Cheng Kung University Tainan Taiwan; ^2^ Institute of Behavioral Medicine, College of Medicine, National Cheng Kung University Tainan Taiwan; ^3^ Clinical Medicine Research Center, National Cheng Kung University Hospital, College of Medicine, National Cheng Kung University Tainan Taiwan; ^4^ Department of General Surgery National Cheng Kung University Hospital, College of Medicine, National Cheng Kung University Tainan Taiwan; ^5^ Department of Surgery Chang Bing Show Chwan Memorial Hospital Changhua Taiwan; ^6^ Asian International Thyroid Center, Chang Bing Show Chwan Memorial Hospital Changhua Taiwan; ^7^ Department of Psychiatry Tainan Hospital, Ministry of Health and Welfare Tainan Taiwan

Mental health issues are recognized among patients with Graves' disease (GD), a cause of hyperthyroidism. The role of the glutamatergic system in major depressive disorder has been confirmed, and glutamatergic agents are considered the next generation of antidepressants.^1^, ^2^ Current drugs include broad glutamatergic modulators (e.g., esketamine), glycine site modulators (e.g., rapastinel and sarcosine), and subunit (NR2B)‐specific *N*‐methyl‐d‐aspartate receptor antagonists (e.g., traxoprodil).^2^ Notably, elevated glutamate levels are related to mania.^3^ Little is known about whether the level of brain glutamate is associated with depression as well as anxiety and mania symptoms among patients with GD, which could be an urgent issue as the use of glutamatergic agents for the treatment of depression has increased.

A total of 26 GD patients with mood and anxiety symptoms (age = 41.19 ± 10.50 years; 22 females and 4 males) were enrolled. The inclusion and exclusion criteria were identical to those of our previous study.^4^ The 17‐item Hamilton Depression Rating Scale (HDRS), the 11‐item Young Mania Rating Scale (YMRS), and the 14‐item Hamilton Anxiety Rating Scale (HARS) were used.

We employed a 3.0 Tesla MRI scanner (GE Discovery MR750, GE Medical Systems, Milwaukee, WI, USA) with an eight‐channel head coil. 1H‐magnetic resonance spectroscopy (1H‐MRS) was performed using point‐resolved spectroscopy (echo time (TE) = 35 ms, repetition time (TR) = 2000 ms, spectral width = 2500 Hz, 2048 data points, 128 water‐suppressed, 16 water‐unsuppressed averages, and eight excitation numbers). The voxels were placed in the medial prefrontal cortex (MPFC; voxel size = 10.0 mL) and the bilateral anterior cingulate cortex (ACC; voxel size = 10.0 mL). Spectra were preprocessed using FID‐Appliance (https://github.com/CIC-methods/FID-A), and concentrations of neurometabolites were measured using linear combination (LC) models. MRS data were fitted and analyzed using the LC Model (version 6.3‐1K). Project‐R version 4.3.2 was used to conduct the correlational analysis and plotting. As the data were not normally distributed, Spearman's *ρ* was used as a conclusive test. The associations between glutamate concentrations and symptoms were probed after controlling for the effects of sex and age via supplemental regression analysis.

A nonparametric Spearman's correlation test indicated that the glutamate concentration in the ACC was associated with the HDRS (glutamate: *ρ* = −0.48, *p* < 0.05; glutamate/creatine: *ρ* = −0.55, *p* < 0.01), while the glutamate concentration in the MPFC was associated with the YMRS (glutamate: *ρ* = 0.67, *p* < 0.001; glutamate/creatine: *ρ* = 0.42, *p* < 0.05), as shown in Figure 1. The significant associations for the absolute level remained robust after controlling for age and sex. No associations were found between the HARS score and glutamate concentration.

**FIGURE 1:**
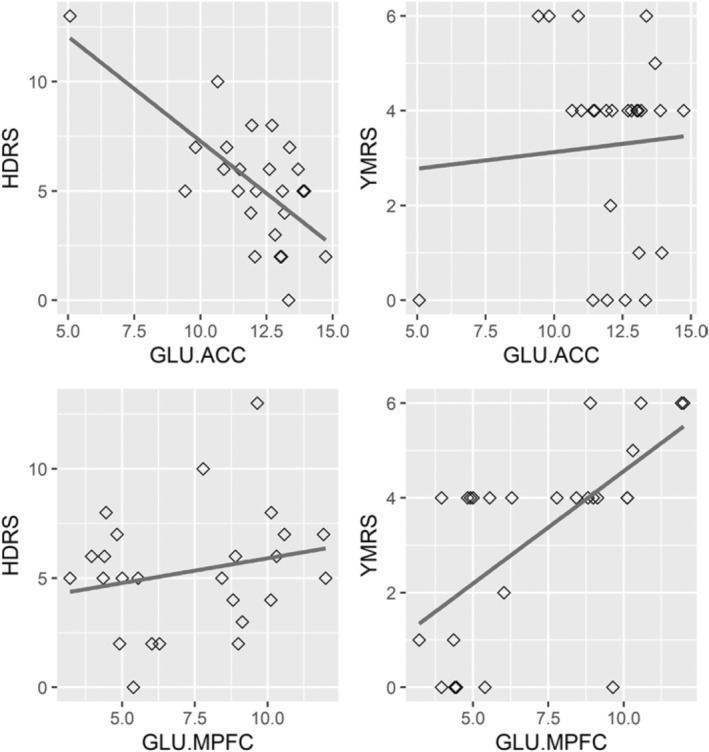
The glutamate concentration in the anterior cingulate cortex was positively related to depression tendency (*ρ* = −0.48, *p* < 0.05; top‐left panel) but not to mania tendency (top‐right panel), while the glutamate concentration in the medial prefrontal cortex was related to mania tendency (*ρ* = 0.67, *p* < 0.001; bottom‐right panel) but not to depression tendency (bottom‐left panel). GLU.ACC, glutamate concentration in the anterior cingulate cortex; GLU.MPFC, glutamate concentration in the medial prefrontal cortex; HDRS, Hamilton Depression Rating Scale; YMRS, Young Mania Rating Scale.

The finding that lower levels of glutamate and more depressive symptoms occur in GD patients is in agreement with the recent glutamate hypothesis of depression, which suggests that the glutamatergic system is also involved in the pathophysiology of depression. This hypothesis has been supported by studies indicating that glutamate levels are lower within the cortex of patients with depression.^5^ The increases in manic symptoms and glutamate concentration could support previous scarce findings.^3^ Currently, little is known about the biological mechanisms involved in the glutamate system in GD patients with mood and anxiety symptoms. Previous findings regarding elevated glutamate levels among mania patients involved the dorsolateral prefrontal cortex.^3^ We speculate that the tendency toward depression and mania in GD patients with certain psychiatric disorders could be related to both higher and lower glutamate levels in the brain. However, because of the heterogeneity of the distribution shown in the figure, the implications of this finding should be considered preliminary. Whether the tendency toward depression and mania among these patients could be influenced by increasing or decreasing glutamate levels in the brain remains to be confirmed by future trials. This phenomenon may imply that intervention in depression among patients with GD via glutamatergic agents should be conducted with caution.

This study had two limitations. First, the causal relationship cannot be confirmed by the cross‐sectional design. Second, the statistical power was limited by the small sample size and the limited distribution of the data.

## CONFLICT OF INTEREST STATEMENT

The authors declare no conflict of interest.
